# A Belief Rule Based Expert System to Assess Tuberculosis under Uncertainty

**DOI:** 10.1007/s10916-017-0685-8

**Published:** 2017-01-30

**Authors:** Mohammad Shahadat Hossain, Faisal Ahmed, Fatema-Tuj-Johora, Karl Andersson

**Affiliations:** 1 Department of Computer Science and Engineering, University of Chittagong, Chittagong, Bangladesh; 2 Pervasive and Mobile Computing Laboratory, Luleå University of Technology, SE-931 87 Skellefteå, Sweden

**Keywords:** Expert system, Belief rule base, Uncertainty, Tuberculosis, Signs and symptoms

## Abstract

The primary diagnosis of Tuberculosis (TB) is usually carried out by looking at the various signs and symptoms of a patient. However, these signs and symptoms cannot be measured with 100 % certainty since they are associated with various types of uncertainties such as vagueness, imprecision, randomness, ignorance and incompleteness. Consequently, traditional primary diagnosis, based on these signs and symptoms, which is carried out by the physicians, cannot deliver reliable results. Therefore, this article presents the design, development and applications of a Belief Rule Based Expert System (BRBES) with the ability to handle various types of uncertainties to diagnose TB. The knowledge base of this system is constructed by taking experts’ suggestions and by analyzing historical data of TB patients. The experiments, carried out, by taking the data of 100 patients demonstrate that the BRBES’s generated results are more reliable than that of human expert as well as fuzzy rule based expert system.

## Introduction

Tuberculosis (TB) is considered as one of the life threatening infectious diseases all over the world, usually, caused by the bacterium Mycobacterium tuberculosis. It is usually two types, namely Pulmonary TB (PTB) and Extra-pulmonary TB (ETB). PTB affects lungs, while ETB can attack any organ of the body except brain, spine, heart, pancreas, skeletal striated muscle, and thyroid. The rate of occurrence of PTB is much higher than that of ETB [1–3].

In 2014, about 9.6 million people became ill and 1.5 million died from TB all over the world. It has been observed that over 95 % death from TB occurs in low and middle income countries. It is considered as one of the top five causes of death for women aged between 15 to 44 [4]. The TB bacteria are usually encapsulated as tiny capsules, called tubercles, in the people with healthy immune system. This stage is known as latent TB.

In this stage, the bacteria remain inactive and cannot spread to other people. On the contrary, when people’s immune system becomes weak and hence, it is unable to prevent the growth of bacteria. Eventually, TB becomes active in the human body. Only active pulmonary TB is contagious and the bacteria spread into the air through the cough and sneeze of the affected people. However, ETB is not contagious. In case of PTB nearby people can easily be infected during inhaling. TB can be fatal if it is not treated in time, causing serious complications in the lungs, forming hole between the nearby airways, making breathing difficult because of blocked airways. The primary signs and symptoms of TB are coughing more than three weeks, coughing up blood, fatigue, unintentional weight loss, chest pain, prolonged fever, lack of appetite and night sweating [1–3].

A physician generally determines the suspicion of TB based on these signs and symptoms. Signs are measured by physician while symptoms are expressed by the patients [5, 6]. Patients usually express the symptoms by using linguistic terms such as high, medium and low, which are imprecise, ambiguous and vague. Therefore, these linguistic terms cannot express the level of symptoms with 100 % certainty and hence, it inherits the types of uncertainty mentioned.

In some cases, patients may ignore the importance of coughing since they consider it is related to other common diseases, which is an example of uncertainty due to ignorance. The sputum smear microscopy, which is a method to diagnose the presence of active TB, sometimes it is unable to detect. This is an example of uncertainty due to incompleteness. A comprehensive survey has been carried out in consultation with the physicians of the various TB hospitals, located in Chittagong District of Bangladesh, to identify the types of uncertainties, associated with each of the signs and symptoms of TB, which are described in Table 1.

**Table 1:** Types of uncertainties associated with tuberculosis

Sign	Types of uncertainty	Description
Coughing	Ignorance	Most patients ignore coughing since they consider it is related to a common disease
	Imprecision	Most patients ignore coughing since they consider it is related to a common disease
	Incompleteness	“Sputum smear microscopy” is the primary method to diagnose pulmonary TB. However, the method is unable to detect half of all active TB cases
Coughing up blood	Ignorance	Sometimes blood remains unnoticed due to thick and larger amount of phlegm
Fatigue	Imprecision, vagueness, ambiguity	People consider fatigue as a normal symptom of overworking and do not consult with doctor
Prolonged fever	Ignorance, vagueness, randomness	Usually patients can not exactly tell the duration and temperature when doctor asks about the fever
Night sweating	Ignorance, vagueness	Most people think that warm weather, humidity or wearing heavy cloths are liable for night sweating and hide the symptom from the doctor

Since the traditional way of determining suspicion of TB is usually carried out by the physicians by looking at the signs and symptoms, it does not consider the above uncertain phenomenon. Thus, the method jeopardizes the accuracy of the detection of TB. However, an expert system which emulates the decision making process of human being can be considered as an appropriate tool to address the uncertain phenomenon to accurately detect the suspicion of TB. An expert system consists of two components, namely knowledge-base and the inference mechanisms. However, such an expert system should have the knowledge representation schema to acquire uncertain clinical knowledge. At the same time, inference mechanism should have the robust reasoning algorithms with the capability to handle various types of uncertainties as mentioned. Therefore, in this study the development of a belief rule-based expert system has been considered, where belief rule base used to handle uncertain knowledge and the evidential reasoning is used as the inference mechanism.

The remaining of the article is organized as follows. Section “Literature review” presents the literature review, “An overview of belief rule based expert system’s methodology” gives an overview of the belief rule based expert systems methodology, and “BRBES to diagnose TB” describes the design, architecture, knowledge-base construction along with an overview of the system interface. Section “Results and discussion” includes the results and discussion, while “Conclusion” concludes the article.

## Literature review

Several studies have been conducted with reference to the diagnosis of Tuberculosis (TB). Multilayer neural networks (MLNN) structures were used to facilitate the analysis of tuberculosis [7]. Back-propagation with momentum and Levenberg-Marquardt algorithms were used to perform the task of training in the MLNN. However, in this approach, an absence of explicit relationship between the input and output data noticed, which is necessary to ensure the transparent diagnosis of TB. Moreover, this approach has not considered the uncertainty issues, associated with the signs and symptoms, related to the input data.

The ensemble classifiers such as Bagging, AdaBoost and Random Forest tree were used to estimate the performance in detecting pulmonary tuberculosis [8]. Coughing sound detection algorithm [9] and lung auscultation software [10] were also used to TB diagnosis. The above approaches considered most of the signs and symptoms as appear in Table 1 in diagnosing TB. However, they lack the procedures to measure these signs and symptoms by taking account of the various uncertainties; rather there measuring approach is Boolean in nature.

In [11], K-mean clustering was combined with the different classifiers, namely, Support Vector Machine (SVM), Naïve Bayesian Classifier and K-Nearest Neighbor (K-NN) to support the prediction of tuberculosis [5].

Moreover, there are systems developed to accurately classify tumor and epilepsy using Genetic Algorithm by combining multiclass classification method and Support Vector Machine (SVM) [12, 13].

However, SVM lacks the transparency [14, 15] since by using this method the relationship between the signs and symptoms of the patient and the diagnosis cannot be established in an understandable way. However, in case of TB diagnosis, a continuous generation of scenario to establish the relationship between the signs and symptoms and the treatment as well as with the diagnostic process is essential [16, 17].

Fuzzy Cluster Means (FCM or Fuzzy C-Mean) analysis [18, 19] used a clustered data set for identifying different types of TB. TUBERDIAG [20] is also a fuzzy rule based system for detecting TB which combines positive and negative knowledge. Although fuzzy based expert system can handle uncertainties due to vagueness, imprecision and ambiguity, they are not equipped to address uncertainties due to ignorance, incompleteness and randomness, which are the cases with the signs and symptoms of TB as shown in Table 1.

In addition, the above mentioned approaches [7–11, 14–20] lack the appropriate inference procedures to address different types of uncertainties such as vagueness, imprecision, ambiguity, ignorance, incompleteness and randomness in a single integrated framework. Such an integrated framework has an important role to make robust decision to support TB diagnostic decision making process.

Therefore, it is necessary to employ an appropriate knowledge representation schema to capture uncertain knowledge that exists with the signs and symptoms of TB. Belief Rule Base (BRB) is widely used to represent this type of knowledge [21–23]. In addition, BRB can be used to demonstrate the explicit non-linear relationship between the input-output data which is necessary to ensure the transparent diagnosis of TB. Evidential reasoning in combination with the BRB can be used as the inference mechanism of the expert system which has the capability to handle all types of uncertainties in an integrated framework [20–28]. Therefore, the following section will represent the belief rule based expert systems (BRBESs) methodology, consisting of knowledge-base construction and the inference procedures.

## An overview of belief rule based expert system’s methodology

An expert system is mainly consists of two components, namely, knowledge-base and the inference procedures. In BRBESs, belief rule-base is used to represent the domain knowledge under uncertainty. On the other hand, inference procedures of BRBEs consists of input transformation, rule activation weight calculation, belief update and rule aggregation using evidential reasoning [21]. Each of them will be elaborated below.

### Domain knowledge representation using BRB

A belief rule is the extension of traditional IF-THEN rule, where a belief structure is used in the consequent part. Antecedent part of the belief rule consists of one or more antecedent attributes with associated referential values, while consequent part consists of one consequent attribute. Knowledge representation parameters such as rule weight and antecedent attribute weight are used. A belief rule base (BRB) consists of one or more than one belief rules. The reason for adopting IF-THEN rule is that it is considered as an appropriate mechanism to represent human knowledge [21].

In addition, non-linear causal relationship between the antecedent and consequent attributes can be established in a belief-rule-base. Equation 1 represents an example of belief rule.

1 $$ R_{k}:\! \left\{\begin{array}{l} \text{IF} \\ (A_{1} \text{ is } A_{1}^{k} )\wedge(A_{2} \text{ is } A_{2}^{k} )\wedge...\wedge(A_{T_{k}} \text{ is } A_{T_{k}}^{k} ) \\ \text{THEN} \\ \left\{\begin{array}{l} \{(C_{1},\overline\beta_{k_{1}}),(C_{2},\overline\beta_{k_{2}}),...,(C_{N},\overline\beta_{k_{N}} )\},((\sum\limits_{n=1}^{N}\overline\beta_{k_{n}}\leq1)), \\ \text{with rule weight } 0 \leq \theta_{k} \leq 1, \\ \text{and attribute weight } {\delta_{1}^{k}},{\delta_{2}^{k}},...,{\delta_{T}^{k}}\geq0 \\ \text{satisfying} \sum\limits_{i=1}^{T_{k}}{\delta_{i}^{k}}=1 \end{array}\right. \end{array}\right. $$

where, $A_{1}, A_{2}, ..., A_{T_{k}}^{k}, T_{k} \in \{1, 2, ..., T\},$ are the antecedent attributes used in the *k*th rule. ${A_{i}^{k}}\in \{A_{i_{1}}, A_{i_{2}}, ..., A_{ij_{i}}\}$ is the referential value of antecedent attribute. *C*_1_, *C*_2_, ..., *C*_*N*_ are the referential values of the consequent attribute while *β*_*ki*_ is the belief degree to which *C*_*i*_ is believed to be true. If $\sum \limits _{i=1}^{N} \overline \beta _{ki} = 1,$ the belief rule is said to be complete, otherwise it is incomplete. In this way, BRB addresses the uncertainty resulting from the incompleteness.

Equation 2 represents the example of a belief rule from the domain of TB. Here, the consequent attribute is “TB Suspicion” with three referential values consisting of “High”, “Medium”, and “Low” with degree of belief 0.8, 0.2, and 0.0. The rule is said to be complete since the summation of the belief degrees stands at 1.

2 $$ \left\{\begin{array}{l} \text{IF} \\ \left\{\begin{array}{l} (\text{Coughing more than 3 weeks is medium) and} \\ \text{(Coughing up blood is High) and} \\ \text{(Chest pain is High) and} \\ \text{(Fatigue is Low) and} \\ \text{(Prolonged fever is medium) and} \\ \text{(Lack of appetite is Low) and} \\ \text{(Weight loss is High) and} \\ \text{(Night sweating is Low)} \\ \end{array}\right. \\ \text{THEN} \\ \text{TB suspicion is (High, 0.8), (Medium, 0.2), (Low, 0.0)} \end{array}\right. $$

Each antecedent attribute of this rule also consists of three referential values and hence, the total number of rules in this BRB can be calculated by applying Eq. 3, which is 6,561.

3 $$ L=\prod\limits_{i=1}^{T}(J_{i}) $$

where *L* is the total number of rules in a sub rule base and *J*_*i*_ is the referential values of the *i*th antecedent attribute.

### BRBESs inference procedures

Each of the inference procedures used in a BRBES is described below.

#### Input transformation

Input transformation consists of the distribution of the input value of an antecedent attribute over its different referential values by applying Eq. 4. The distributed value with each referential values of the antecedent attribute is called matching degree or the degree of belief. It is interesting to note that when the value of this matching degree is calculated for each of the referential values of the antecedent attribute (AA), this value is assigned to only those rules where this referential value exits with the AA.

As an example, Eq. 2 consists of eight antecedent attributes, each with three referential values. When the input data for one antecedent attribute is collected from the TB patient, its matching degrees to the corresponding referential values are calculated by applying Eq. 4. Consequently, the matching degree, related to a referential value corresponding to the antecedent attribute of a rule is assigned. In this way, input data for the eight antecedent attributes can be collected for a patient and their corresponding matching degree is assigned in a rule. Once the rule is assigned with the corresponding matching degree then it is said to be active and the rule is called packet antecedent. This phenomenon can be described that the rule is in the RAM while the initial rule base is in the secondary memory.

4 $$ \alpha_{ih}\,=\,\frac{u(A_{ih+1})-A_{i}^{*}}{u(A_{ih+1})-u(A_{ih})} \text{ and } \alpha_{ih+1}=\frac{A_{i}^{*}-u(A_{ih})}{u(A_{ih+1})-u(A_{ih})} $$

where *u*(*A*_*ih*+1_) and *u*(*A*_*ih*_) are grade values of *A*_*ih*+1_ and *A*_*ih*_ respectively.

Table 2 shows the matching degree of the antecedent attribute value into its different referential values. For example, the input value of the antecedent attribute ”Cough” is identified as ”low”, which is weighted as 10 % by the expert and its corresponding matching degrees associated with the referential values (in this case they are “High”, “Medium” and “Low”) obtained by using Eq. 4.

**Table 2:** Input Transformation

Serial No.	Antecedent Name	Antecedent Value	High	Medium	Low
1	Cough	Low, 10 %	0	0.2	0.8
2	Blood with cough	High, 60 %	0.2	0.8	0
3	Chest pain	High, 80 %	0.6	0.4	0
4	Fatigue	Low, 30 %	0	0.6	0.4
5	Fever	High, 85 %	0.7	0.3	0
6	Lack of appetite	Medium, 50 %	0	1	0
7	Weight loss	High, 90 %	0.8	0.2	0
8	Night sweating	Low, 15 %	0	0.3	0.7

#### Calculation of activation weights

Rule activation weight calculation consists of calculating the combined matching degree (*α*_*k*_) as well as the weight of a rule in the BRB. Equation 2 consists of eight antecedent attributes and hence, it is important to calculate their combined matching degree, which can be calculated by using multiplicative function [as shown in Eq. 5], allowing the inter-relationship between the attributes [21].

5 $$ \alpha_{k}=\prod\limits_{i=1}^{T_{k}}({\alpha_{i}^{k}})^{\overline{\delta}_{i}^{k}}, \overline{\delta}_{i}^{k}=\frac{{\delta_{i}^{k}}}{\smash{\displaystyle\max_{i=1, ..., T_{k}}({\delta_{i}^{k}})}} $$

so that $0 \leq {\delta _{i}^{k}} \leq 1$ where ${\delta _{i}^{k}} (i=1, ..., T_{k})$ is the relative weight of the *i*th antecedent attribute in the *k*th belief rule. *T*_*k*_ is the total number of antecedent attributes in the *k*th rule. Here, ${\delta _{i}^{k}}$ = 0 is meaning that the attribute which has zero importance and hence, no impact on the aggregation process, while ${\delta _{i}^{k}}$ = 1 demonstrates the significant impact. Moreover, overall belief increases with the increment of the individual belief of the antecedent attributes. The rule activation weight is calculated by using Eq. 6 [21].

6 $$ W_{k}=\{\theta_{k}\alpha_{k}\}\left\{\sum\limits_{i=1}^{L}\theta_{i}\alpha_{i}\right\} $$

where *𝜃*_*k*_ is the relative weight of the *k*th rule and *L* is the total number of belief rule in the belief rule-base. When the *W*_*k*_ of a rule is zero then it has no impact in the BRB while it is “1” then its important is high.

#### Belief degree update

Equation 2 shows the presence of eight antecedent attributes, necessary to assess the suspicion of TB. However, there could be some situation when the data of the some attributes could not be available. In such situation, the initial belief degrees that were assigned to the referential values of the consequent attribute needs to be updated by applying Eq. 7 [21]. This phenomenon is an example uncertainty due to ignorance.

7 $$ \beta_{ki}=\overline{\beta}_{ki} \frac{{\sum}_{t=1}^{Tk}(\tau(k,t){\sum}_{j=1}^{J_{i}}\alpha{}t_{j})}{{\sum}_{t=1}^{T_{k}}\tau(k,t)} $$

where *τ*(*k*,*t*)= $\left \{\begin {array}{lll}1, \text { if used in defining } R_{k}(t=1, ..., T_{k}) \\ \text {or} \\ 0, \text { otherwise} \end {array}\right .$

Here, $\overline {\beta }_{ki}$ is the original belief degree while *β*_*ki*_ is the updated belief degree. The original belief degree is updated while any ignorance is noticed. For example, if the antecedent “cough” is ignored, then the initial belief degrees are updated as shown in Table 3.

**Table 3:** Belief Degree Update

Rule Id		High	Medium	Low
2	Initial	0	0.6	0.4
	Update	0	0.48	0.32

#### Inference using evidential reasoning

In order to obtain the aggregated value of the referential values of the consequent attribute, based on the input data of the antecedent attributes, either recursive or analytical evidential reasoning (ER) algorithms as shown in Eq. 8 can be applied [22]. To reduce the computational complexity analytical ER algorithm is found effective to calculate the final belief degree *β*_*j*_.

8 $$ \beta_{j}=\frac{\mu\times[{\prod}_{k=1}^{L}(\omega_{k}\beta_{kj}+1-\omega_{k}{\sum}_{j=1}^{N}\beta_{kj})-{\prod}_{k=1}^{L}(1-\omega_{k}{\sum}_{j=1}^{N}\beta_{kj})]}{1-\mu{}\times{}[{\prod}_{k=1}^{L}(1-\omega_{k})]} $$

with $\mu = [{\sum }_{j=1}^{N}{\prod }_{k=1}^{L}(\omega {}_{k}\beta {}_{kj}+1-\omega _{k}{\sum }_{j=1}^{N}\beta {}_{kj})-(N-1)\times {\prod }_{k=1}^{L}(1-\omega _{k}{\sum }_{j=1}^{N}\beta _{kj})]^{-1}$

The final combined result or output generated by ER is represented by {(*C*_1_,*β*_1_),(*C*_2_,*β*_2_),...,(*C*_*N*_,*β*_*N*_)} where *β*_*j*_ is the final belief degree attached to the *j*th referential value *C*_*j*_ of the consequent attribute *C*, which is obtained after all activated rules in the BRB are combined by using ER. This output can be converted into a crisp/numerical value [as shown in Eq. 9] by assigning a utility score to each referential value of the consequent attributes [20].

9 $$ H(A^{*})=\sum\limits_{j=1}^{N}u(C_{j})\beta_{j} $$

where *H*(*A* ^∗^) is the expected score expressed as a numerical value and *u*(*C*_*j*_) is the utility score of the *j*th referential value.

## BRBES to diagnose TB

This section presents the architecture along with implementation strategy of the belief rule-based expert system (BRBES) to diagnose Tuberculosis (TB). This is followed by the presentation of the knowledge-base construction as well as a description of the BRBES’s interface.

### Architecture and implementation

**Fig. 1:**
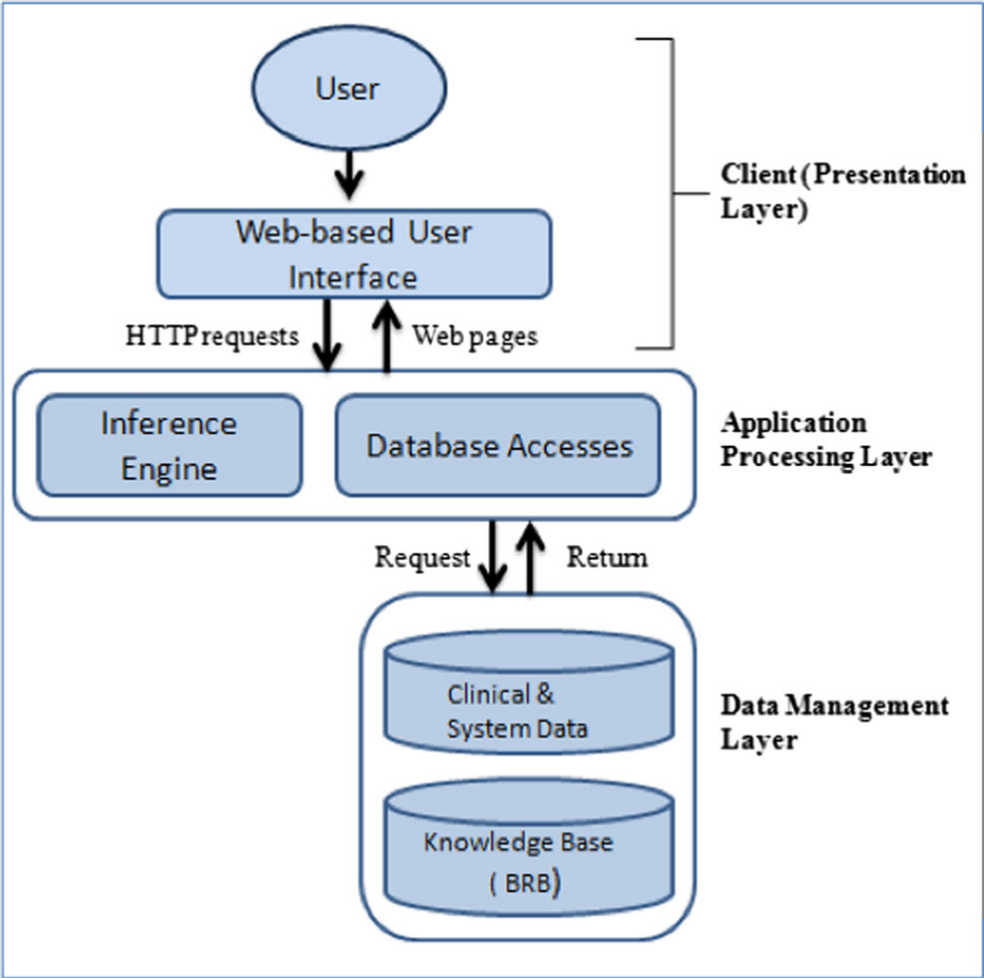
Architecture of BRBES

A system architecture can be defined how its components are organized. It is also important to know the pattern of system organization, which is known as architectural style. BRBES presented in this article follows three-layer architecture, consisting of web-based interface, application and database management layers as shown in Fig. 1.

Web-based interface: Since the BRBES to be used by the physicians, patients and researchers at various hospitals of Bangladesh, especially in the rural areas, a web-based user-friendly interface is necessary. Therefore, to ensure the usability of the system by the mentioned users at any time and at any place, a web-based interface has been developed for the BRBES. This interface has been developed by integrating various web technologies such as Javascript, Jquery, HTML and CSS. The application layer, which facilities inference and database access, has been developed by using PHP because of its simplicity, shorter development cycle and it can be used through online. The data-base management layer, which consists of clinical data and knowledge-base, developed by using MySQL, which is a relational database management system. MySQL is exible, user friendly and ensures security as well as faster data access.

### Knowledge base construction in BRB

**Fig. 2:**
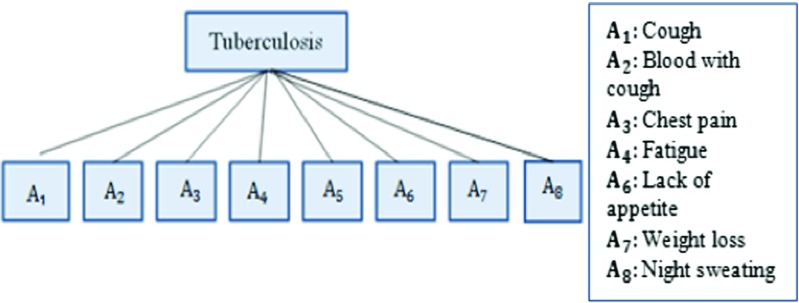
BRB Framework to Assess TB

The knowledge-base construction consists of developing a BRB tree by identifying the necessary antecedent and consequent attributes. Figure 2 shows the single level BRB structure to diagnose TB. The leaf nodes represent the antecedent attributes while root node consequent attribute. The eight antecedent attributes, having three referential values each, are identified and they are verified in consultation with the physicians, located at the various hospitals of Chittagong City of Bangladesh.

The BRB consists of 6,561 rules since it comprises eight antecedent attributes each with three referential values. The number of rules are calculated by using Eq. 3. A BRB usually can be established [20] by acquiring domain expert knowledge, by collecting historical data, by using earlier rules if they are available and by developing rules in a random way without any prior-knowledge.

In this study, the initial BRB has been constructed by acquiring knowledge of physicians or the domain experts as well as by using patients’ historical data. Each rule of the BRB has given rule weight “1” while each antecedent attribute’s weight is considered as “1”. An example of such rule is illustrated in Eq. 2. Table 4 illustrates the initial BRB of the BRBES.

**Table 4:** A Sample of Initial Belief Rule-Base for Assessment of Tuberculosis Suspicion

Rule No	Rule Weight	Antecedents								Consequent		
		IF								Then		
		A1	A2	A3	A4	A5	A6	A7	A8	TB suspicion		
										High	Medium	Low
1	1	H	H	H	H	L	H	H	H	1.0	0.0	0.0
2	1	H	L	M	L	H	L	L	L	0.0	0.6	0.4
3	1	L	H	L	M	H	L	L	M	0.0	0.1	0.9
4	1	L	L	L	L	H	L	L	L	0.0	0.0	1.0
5	1	H	M	H	L	H	L	L	H	0.1	0.5	0.4
6	1	M	H	H	L	M	L	H	L	0.8	0.2	0.0
7	1	H	M	M	M	H	L	L	M	0.1	0.7	0.2
8	1	M	L	L	L	H	M	M	L	0.1	0.6	0.3
9	1	H	H	H	H	H	H	H	M	0.4	0.6	0.0
10	1	L	M	H	H	H	M	M	L	0.0	0.1	0.9

### BRB interface

**Fig. 3:**
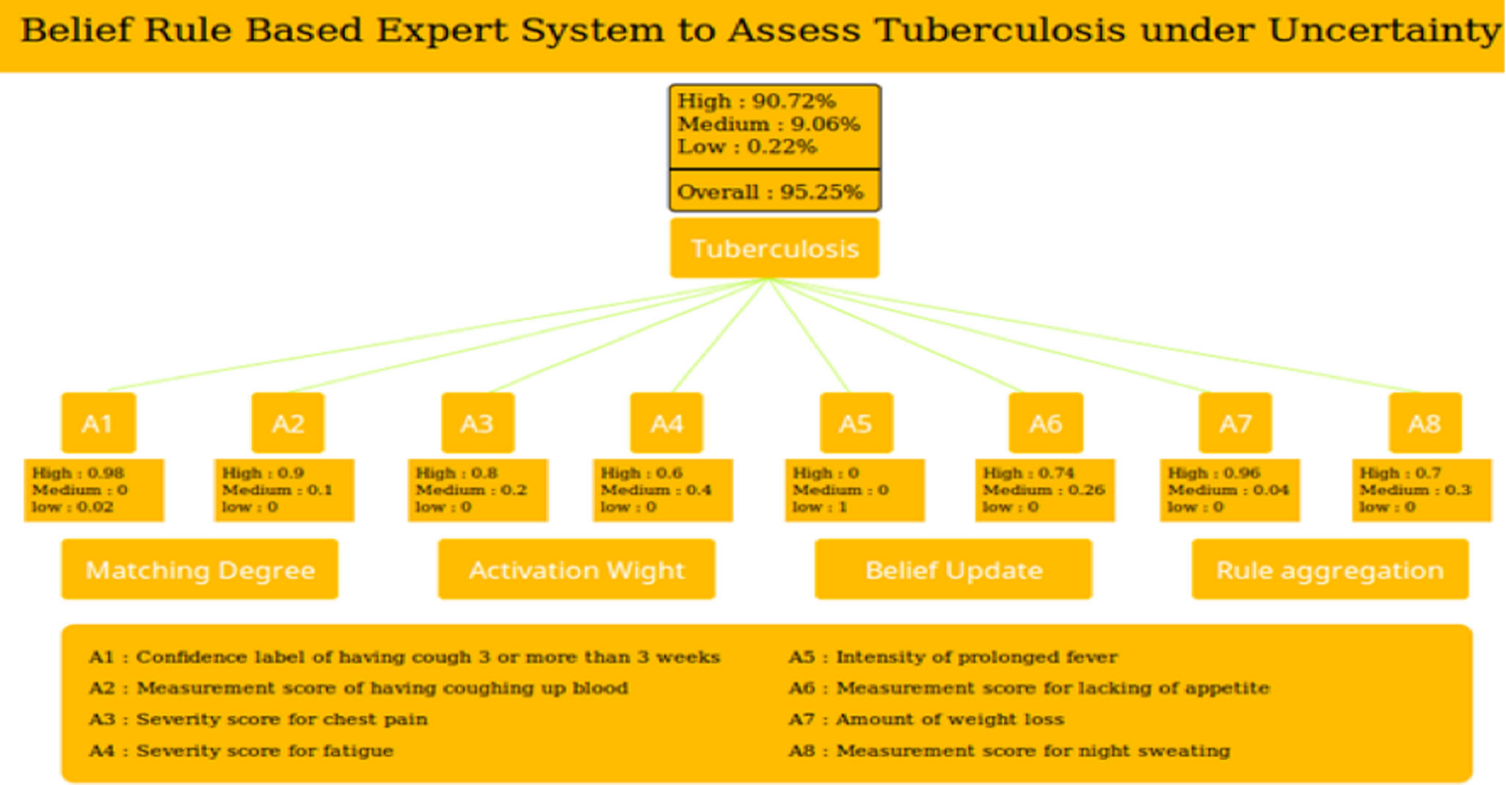
BRBES interface to assess TB

An interface can be defined as a media, facilitating the users to interact with the system. Figure 3 illustrates the interface of the BRBES to diagnose TB, allowing the acquisition of the input value, associated with each of the eight antecedent attributes, either from the patients or from the physicians. Figure 3 illustrates the matching degree, associated with the antecedent attributes with reference to the data of the first patient as shown in Table 5.

**Table 5:** TB Suspicision by Fuzzy Logic, BRBES, and Expert

SL. NO (1)	A1 (2)	A2 (3)	A3 (4)	A4 (5)	A5 (6)	A6 (7)	A7 (8)	A8 (9)	Fuzzy Result(%) (10)	BRBES Result(%) (11)	Expert Opinion(%) (12)	Bench- mark (13)
1	98	95	90	80	98	87	9.8	85	76.34	95.25	70	1
2	95	15	45	22	103	30	1.0	15	54.72	51.64	52	1
3	25	95	15	55	102.5	20	1.8	60	58.71	42.93	55	0
4	10	30	27	18	101.5	25	3.5	12	32.67	19.47	45	0
5	18	60	87	12	99.5	15	1.5	80	38.81	33.28	41	1
6	90	86	98	18	98	17	9.7	23	65.53	72.45	67	1
7	94	58	67	45	102	16	2.5	67	70.77	74.67	70	1
8	85	32	13	20	104	78	1.2	23	49.23	64.13	46	1
9	92	97	75	87	98	96	8.9	55	72.56	91.63	69	1
10	15	67	60	92	101.5	67	1.2	15	45.61	32.01	50	0
11	90	87	69	60	103	75	4	60	73.03	84.05	75	1
12	87	46	82	77	99	68	4	76	84.45	84.77	82	1
13	75	80	90	74	101.5	90	2	83	82.12	85.18	77	1
14	46	70	82	91	104	40	1	75	56.32	69.86	60	1
15	70	80	85	70	103	66	3	68	78.71	80.26	81	1

For example, the input value of the antecedent attribute A1 (coughing more than three weeks) is obtained as “98”. This is acquired by asking the patient about the intensity level of the A1, which is in the range of 0-100. This input value is then distributed over the three referential values of A1, which are “(High, 0.98)”, “(Medium, 0.0)” and “(Low, 0.2)”. This distributed value is called the matching degree obtained by using Eq. 4. The suspicion level of the TB of this patient is obtained by using Eqs. 5, 6, 7 and 8. This is measured as degree of belief associated with each of the referential values of the suspicion level of Tuberculosis and found as (High, 90.72), (Medium, 0.06), and (Low, 0.22). These fuzzy values are converted into crisp value by using Eq. 9 and obtained as 95.25 %.

## Results and discussion

**Fig. 4:**
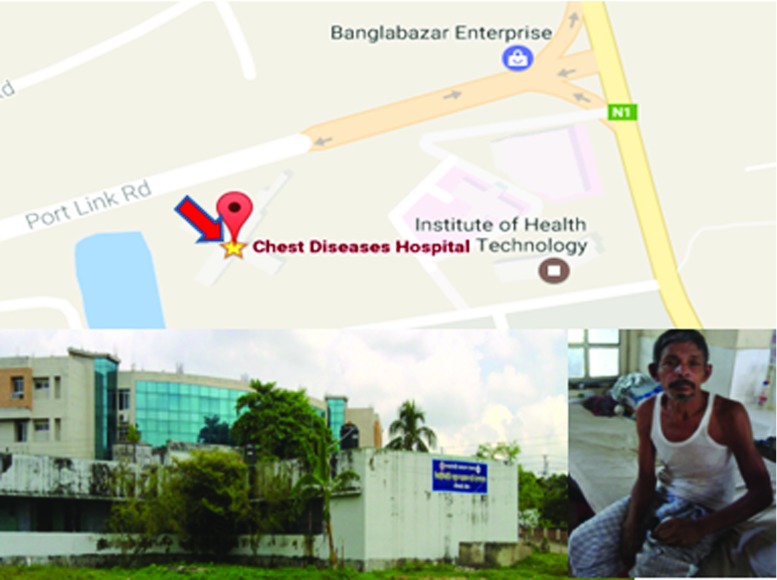
Tuberculosis Hospital, Chittagong, Bangladesh

To demonstrate the applicability and the reliability of the BRBES to diagnose tuberculosis (TB), the system fed with the input data received from the TB patients of a hospital located in the Chittagong City of Bangladesh (Fig. 4).

The input data, associated with the eight attributes, of 77 TB patients have been collected. The real laboratory test results of those patients collected and they were considered as the benchmark data. If TB is found positive in laboratory test result for a patient, then the benchmark is considered as “1”, otherwise it is “0” for that patient. Table. V illustrates the collected data on the eight attributes of a patient (column 2 through 9) along with level of TB suspicion generated by the BRBES (Column 11). Table. V also illustrates the expert opinion on the level of TB suspicion (Column 12) and the benchmark data is recorded in column 13. In order to compare the reliability of the BRBES’s results a Fuzzy Rule Based Expert System (FRBES) to measure the suspicion level of TB developed in MatLab environment and the results generated for the same data by using FRBES are recorded in Column 10. For simplicity, Table 5 only presents the data of 15 patients out of 77.

**Fig. 5:**
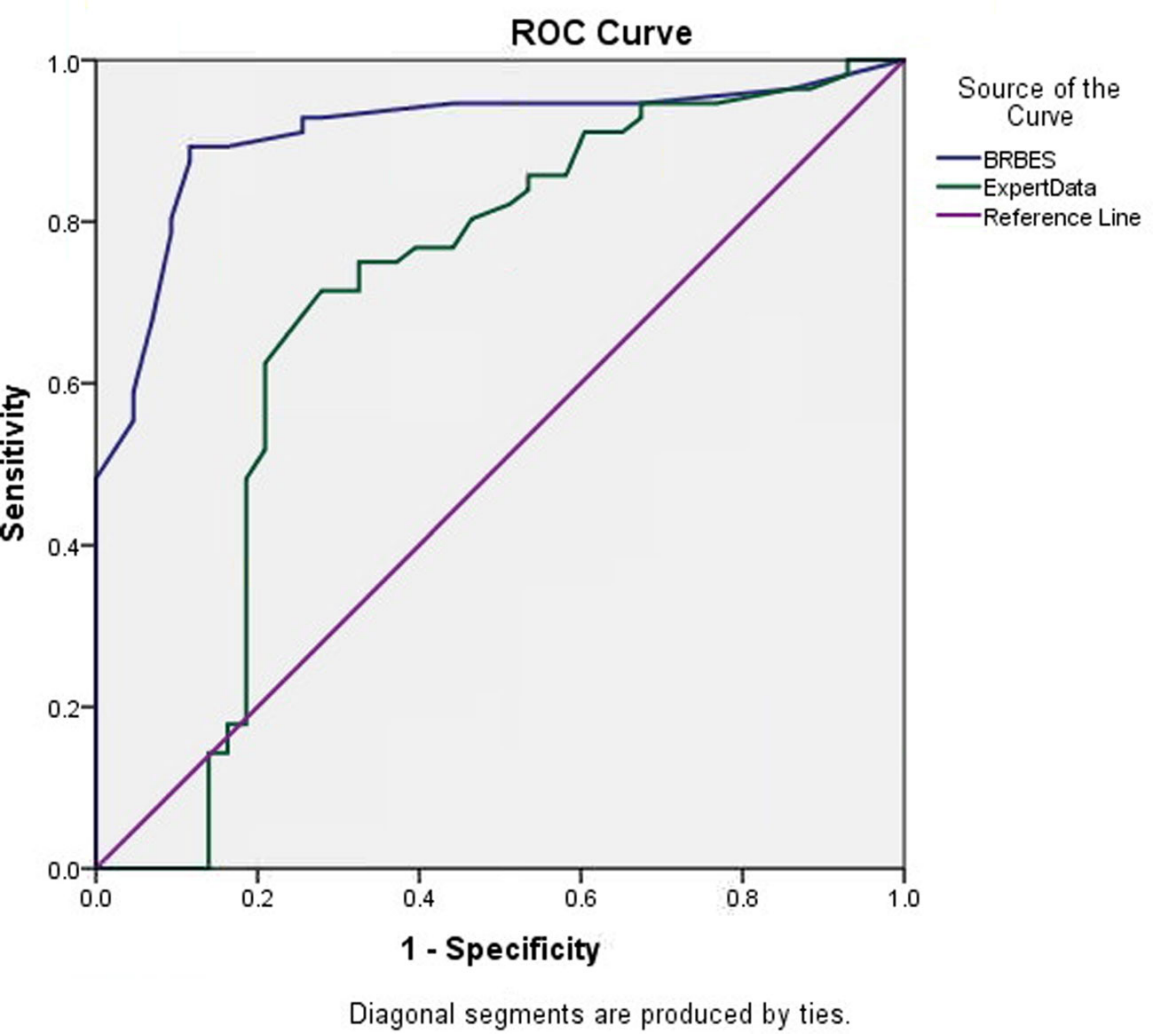
ROC Curves Comparing the result of BRBES and Expert Data

The Receiver Operating Characteristic (ROC) curves are usually used to analyze the accuracy and reliability of the diagnostic tests having ordinal or continuous results. Therefore, the method was considered, to measure the reliability of BRBES in comparison with expert opinion and FRBES. The accuracy of a system to assess the level of suspicion of the TB can be measured by calculating the Area Under Curve (AUC) [29, 30]. For example, if the AUC is found to be one for the results generated by the BRBES then the system can be considered as 100 % reliable. Figure 5 illustrates the ROC curves plotted for both the BRBES and the Expert Opinion. The ROC curve plotted by the blue line in this figure is associated with the results generated by the BRBES with AUC of 0.910 (95 % confidence intervals 0.848 - 0.972).

**Fig. 6:**
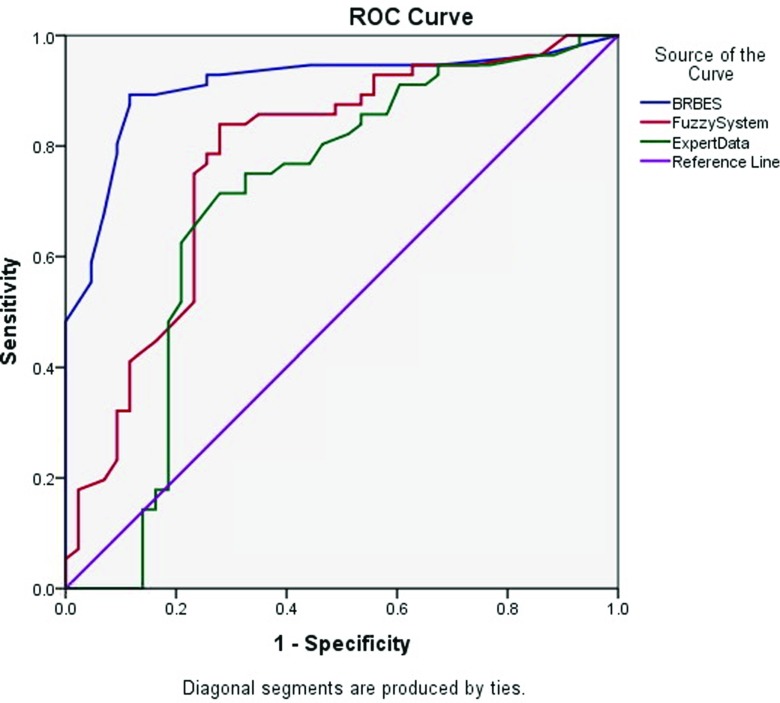
ROC Curves Comparing the Result of BRBES, FRBES and Expert Data

The ROC curve plotted by the green line in Fig. 5 is obtained against the physician’s opinion, and its AUC is 0.710 (95 % confidence intervals 0.587 - 0.815). However, Fig. 6 illustrates the ROC curves for BRBES, FRBES and Expert Opinion.

The ROC curve plotted by the red line in Fig. 6 is obtained against the FRBES and its AUC is 0.777 (95 % confidence intervals 0.680 - 0.873). While the AUC values of Fig. 6 for both the BRBES and the Expert Opinion are same as of Fig. 5. Table 6 summarizes the above results associated with BRBES, FRBES and Expert Opinion. From Figs. 5 and 6 as well as from Table 6 it can be observed that AUC of Expert Opinion is much less than from both the BRBES and FRBES. The reason for this is that during the interviewing and conversation with the physicians it has been observed that they are not aware of the uncertainty issues related to the signs and symptoms of the TB.

**Table 6:** Reliability Comparison among Three Systems

Test Result Variables	Area	Asymptotic 95 % Confidence Interval	
		Lower Bound	Upper Bound
BRBES	0.910	0.848	0.972
Fuzzy System	0.777	0.680	0.873
Expert Data	0.701	0.587	0.815

The reason for this is that during the interviewing and conversation with the physicians it has been observed that they are not aware of the uncertainty issues related to the signs and symptoms of the TB. Therefore, the reliability of their assessment level of TB suspicion is much lower than that of BRBES and FRBES. From the Table 6 as well as from Figs. 5 and 6, it can also be observed that AUC of BRBES is much larger than that of FRBES. The reason for this is that fuzzy rule based expert system (FRBES) only considers uncertainty due to the vagueness, imprecision and ambiguity. However, BRBES includes uncertainties due to the ignorance, incompleteness and randomness in addition to the vagueness, imprecision and ambiguity.

Further, the inference procedures of the FRBES which uses either Mamdanior T-S methods are not equipped to process uncertainty issues during the reasoning process. On the contrary, BRBES uses evidential reasoning procedures as the inference engine which is equipped to handle types of uncertainties mentioned before.

## Conclusion

This article described the design, implementation and applications of a Belief Rule Based Expert system (BRBES), allowing the measurement of the level of suspicion of TB by taking account of its various signs and symptoms. The system allows the understanding of the relationship between signs and symptoms and the level of suspicion of TB of a patient in an explicit and transparent way. This will allow the identification of the signs and symptoms those are responsible for increasing the suspicion level of TB of a patient. In this way, various scenarios by taking account of the signs and symptoms of a patient from various perspectives can be carried out by using the BRBES. The physicians will eventually be able to select the appropriate medicine for a patient. In addition, all types of uncertainties such as vagueness, imprecision, ambiguity, ignorance and incompleteness can be handled in an integrated framework which makes the system more robust as evident from the comparative results generated by using both the manual system and the fuzzy rule based expert system as illustrated in Table 6. Therefore, the BRBES can provide a decision support platform to the physicians and would serve a savior by offering primary health care to the people with reduced time and low diagnosis cost. In future, the BEBES would be extended to support optimal learning by training the knowledge representation parameters such as rule weight, attribute weight, and belief degrees.

## References

[1] Walker, B.R., Colledge, N.R., Ralston, S.H., Penman, I.D., *Davidson’s principles and practice of medicine*. 22nd ed. United Kingdom: Elsevier Health Sciences, 2014.

[2] Goldman L, Bennet JC (2012). Cecil textbook of medicine.

[3] Walker HL, Chen-Yuan C, Gie RP, Enarson DA (2009). Crofton’s clinical tuberculosis.

[4] WHOTuberculosis, http://www.who.int/mediacentre/factsheets/fs104/en/, accessed: January 7, 2017

[5] Karim, R., Hossain, M.S., Khalid, M.S., Mustafa, R., Bhuiyan, T.A.: A belief rule based expert system to assess bronchiolitis suspicion from signs and symptoms under uncertainty. In: SAI Intelligent systems conference (intellisys), p. 2016. UK, London

[6] Hossain MS, Zander P-O, Kamal MS, Chowdhury L (2015). Belief-Rule-Based Expert systems for evaluation of E-Government: a case study. Expert. Syst..

[7] Er O, Temurtas F, Çetin Tannkulu A (2010). Tuberculosis disease diagnosis using artificial neural networks. J. Med. Syst..

[8] Rusdah, E., and Wardoyo, R.: Winarko Preliminary diagnosis of pulmonary tuberculosis using ensemble method. In: International conference on data and software engineering (ICoDSE), p. 2015. Yogyakarta, Indonesia

[9] Tracey, B. H., Comina, G., Larson, S., Bravard, M., López, J.W., Gilman, R.H.: Cough detection algorithm for monitoring patient recovery from pulmonary tuberculosis. In: 2011 Annual international conference of the IEEE engineering in medicine and biology society, EMBC, p. 2011. USA, Boston, MA 10.1109/IEMBS.2011.6091487 22255711

[10] Lestari, R., Ahmad, M., Alisjahbana, B., Djatmiko, T.: The Lung Diseases Diagnosis Software: Influenza and Tuberculosis Case Studies in The Cloud Computing environment. In: 2012 International conference on cloud computing and social networking (ICCCSN), p. 2012. Bandung, Indonesia

[11] Asha, T., Natarajan, S., Murthy, K.N.B., A data mining approach to the diagnosis of tuberculosis by cascading clustering and classification. *J. Comput.* 3(4), 2011.

[12] Shen, C.P., Lin, J.W., Lin, F.S., Lam, A.Y.Y., Chen, W., Zhou, W., Sung, H.Y., Kao, Y.H., Chiu, M.J., Leu, F.Y., Lai, F., GA-SVM Modeling of multiclass seizure detector in epilepsy analysis system using cloud computing. *Soft. Comput.*,1–11, 2015. doi:10.1007/s00500-015-1917-9.

[13] Lin FS, Shen CP, Liu CH, Lin H, Huang CYF, Kao CY, Lai F, Lin JW (2015). A high performance multiclass classification framework using cloud computing architecture. Journal of Medical and Biological Engineering.

[14] Calzada A, Liu J, Wang H, Nugent C, Martinez L (2015). Application of a spatial intelligent decision system on Self-Rated health status estimation. J. Med. Syst..

[15] Huysmans J, Dejaeger K, Mues C, Vanthienen J, Baesens B (2011). An empirical evaluation of the comprehensibility of decision table, tree and rule based predictive models. Decis. Support. Syst..

[16] Ainon RN, Bulgiba AM, Lahsasna A (2012). AMI Screening using linguistic fuzzy rules. J. Med. Syst..

[17] Bojarczuk CC, Lopes HS, Freitas AA (2000). Genetic programming for knowledge discovery in chest pain diagnosis. IEEE Eng. Med. Biol. Mag..

[18] Soundararajan K, Sureshkumar S, Anusuya C (2012). Diagnostics decision support system for tuberculosis using fuzzy logic. International Journal of Computer Science and Information Technology & Security (IJCSITS).

[19] Imianvan, A.A., and Obi, J., Fuzzy cluster means expert system for the diagnosis of tuberculosis. *Global J. Comp. Sci. Technol.* 11(6), 2011.

[20] Phuong, N.H., Hung, D.H., Tuan, D.T., Co, N.V., Duong, B.D., Thinh, P.T., Hoa, N.P., Linh, N.N.: Designing an experimental expert system for lung tuberculosis diagnostics using fuzzy set theory. In: 1998 IEEE International conference on systems, man, and cybernetics, p. 1998. USA, San Diego, CA

[21] Yang J-B, Liu J, Wang J, Sii H-S, Wang H-W (2006). Belief rule-base inference methodology using the evidential reasoning approach-rimer. IEEE Transactions on Systems Man, and Cybernetics-part A: Systems and Humans.

[22] Hossain, M.S., Monrat, A.A., Hasan, M., Karim, R., Buhiyan, A.T., Khalid, M.S.: A belief rule based expert system to assess mental disorder under uncertainty. In: 2016 5th international conference on informatics, electronics & vision (ICIEV), p. 2016. Dhaka, Bangladesh

[23] Hossain, M.S., Andersson, K., Naznin, S.: A belief rule based expert system to diagnose measles under uncertainty. In: Proceedings of the 2015 international conference on health informatics and medical systems (HIMS 2015). Las Vegas, Nevada, USA (2015)

[24] Hossain, M.S., Hasan, M.A., Uddin, M., Islam, M.M., Mustafa, R.: A belief rule based expert system to assess lung cancer under uncertainty. In: 2015 18Th international conference on computer and information technology (ICCIT), p. 2015. Dhaka, Bangladesh

[25] Karim, R., Andersson, K., Hossain, M.S., Uddin, M.J., Meah, M.P.: A belief rule based expert system to assess clinical bronchopneumonia suspicion. In: Future Technologies Conference 2016 (FTC 2016), San Francisco, CA, USA (2016)

[26] Hossain, M.S., Haque, M.A., Mustafa, R., Karim, R., Dey, H.R., Yousuf, M.: An expert system to assist the diagnosis of ischemic heart disease, Int J Integr Care (2016)

[27] Hossain, M.S., Khalid, M.S., Akter, S., Dey, S.: A belief rule-based expert system to diagnose influenza. In: 2014 9Th international forum on strategic technology (IFOST). Cox’s Bazar, Bangladesh (2014)

[28] Rahaman, S., and Hossain, M.S.: A belief rule based clinical decision support system to assess suspicion of heart failure from signs, symptoms and risk factors. In: 2013 International conference on informatics, electronics & vision (ICIEV), p. 2013. Dhaka, Bangladesh

[29] Metz CE (1978). Basic principles of ROC analysis. Semin. Nucl. Med..

[30] Hanley JA (1988). The robustness of the binormal assumptions used in fitting ROC curves. Med. Decis. Mak..

